# Association between *CISD2* expression and clinicopathological features and prognosis in gastric cancer: a systematic review, meta-analysis, and database validation

**DOI:** 10.3389/fonc.2026.1965844

**Published:** 2026-10-20

**Authors:** Tong Liang, Yaqiong Cui, Hongmei Song

**Affiliations:** 1 The First School of Clinical Medicine, Gansu University of Chinese Medicine, Lanzhou, Gansu, China; 2 Department of Radiology, Gansu Provincial Hospital, Lanzhou, Gansu, China; 3 Department of Oncology I, Gansu Provincial Hospital of Traditional Chinese Medicine, Lanzhou, Gansu, China

**Keywords:** CDGSH iron sulfur domain 2, gastric cancer, meta - analysis, prognosis, tumor marker

## Abstract

**Objective:**

This study conducted an exploratory hypothesis-generating investigation to assess the relationship between CDGSH iron sulfur domain 2 (CISD2) and gastric cancer (GC) via meta-analysis supplemented by independent in silico database analysis.

**Methods:**

We systematically searched 7 major databases including Wan Fang, VIP, CNKI, SinoMed, Web of Science, The Cochrane Library, and PubMed from database inception to July 2026 for studies reporting *CISD2* expression data in GC patients. Two researchers independently performed literature screening, data extraction, and quality assessment. Stata 12.0 was used to conduct the meta-analysis of protein-level *CISD2* expression data from immunohistochemistry (IHC) studies. The Kaplan-Meier plotter database was used as an independent complementary analysis to explore the association between *CISD2* transcript-level expression and GC prognosis. The study protocol was registered in PROSPERO (CRD42023490404).

**Results:**

Three high-quality studies with a total of 1318 participants were finally included. The exploratory meta-analysis showed no statistically significant association between *CISD2* protein expression and age, gender, lymphatic invasion, TNM stage, or histological grade in GC patients. A suggestive negative correlation was observed between *CISD2* IHC expression and tumor invasion depth, with a pooled *OR* of 0.57 (95% *CI*: 0.41 - 0.79, *P* = 0.001). The independent transcript-level in silico analysis from the Kaplan-Meier plotter provided complementary prognostic evidence, showing that elevated *CISD2* mRNA expression was significantly correlated with poor first progression (*HR* = 1.3, *P* = 0.04) and reduced overall survival (*HR* = 1.27, *P* = 0.041) in GC patients.

**Conclusion:**

This exploratory study generates preliminary hypotheses that *CISD2* may have a potential association with GC progression and prognosis. Given the limited number of included studies and the distinct data types across analyses, large-scale prospective studies and mechanistic investigations are urgently required to verify these associations before any clinical application of *CISD2* as a biomarker can be established.

## 1 Introduction

Gastric cancer (GC) stands as one of the most prevalent malignancies globally, exerting a substantial toll on worldwide health (1). Although notable progress has been made across various treatment approaches, the prognosis for GC patients remains dismal (2), primarily attributed to late-stage diagnoses and a scarcity of effective therapeutic alternatives (3). Hence, there is an urgent need to identify innovative prognostic biomarkers capable of reliably forecasting clinical outcomes.

CDGSH iron sulfur domain 2 (CISD2) is a gene that has only recently been discovered and holds a pivotal position in regulating oxidative stress responses and maintaining cellular iron homeostasis (4–6). Aberrant *CISD2* expression has been detected in a range of cancers (7–13), with GC being no exception (8–10). However, its prognostic value and clinical relevance in GC are still shrouded in uncertainty.

In this research, we undertook a comprehensive meta-analysis coupled with database validation to elucidate the prognostic significance of *CISD2* in GC. Following an exhaustive search across multiple electronic databases, we incorporated studies that evaluated the association between *CISD2* expression and clinical outcomes in GC patients. Subsequently, we scrutinized these findings using publicly accessible GC datasets, computing pooled hazard ratios (HR) along with their corresponding 95% confidence intervals (CI) to ascertain the influence of *CISD2* on patient survival.

As detection methods for *CISD2* and criteria for assessing immunohistochemical results differ among studies, conclusions may be affected. Hence, this study selected high-quality research that employed immunohistochemistry to measure *CISD2* expression, evaluating it based on both staining pattern and intensity. By conducting a meta-analysis to aggregate data, we strive to pinpoint new molecular targets for the early diagnosis and treatment of GC, offering evidence-based clinical guidance.

## 2 Materials and methods

### 2.1 Systematic literature search

A meticulously crafted and comprehensive search strategy was implemented to systematically identify relevant studies exploring the relationship between *CISD2* and GC. The full reproducible search strategy for PubMed is provided below, with equivalent adapted search terms applied across Wan Fang, VIP, China National Knowledge Infrastructure (CNKI), SinoMed, Web of Science, and The Cochrane Library. The search spanned from the inception of each database through to July 15, 2026, which is set as the final search date of this study. The PRISMA diagram has been revised to correct the prior misclassification of literature sources: the 708 initially identified records were all sourced from the 7 selected electronic databases, no dedicated registry search was performed in this study.

Full PubMed Search Strategy:

(((“CDGSH iron sulfur domain 2”[MeSH Terms]) OR (“CDGSH iron sulfur domain 2”[Title/Abstract])) OR (“CISD2”[Title/Abstract])).

The search methodology seamlessly combined subject-specific MeSH terms and free-text keywords to maximize retrieval accuracy. Reference lists of all identified studies were also scrutinized to unearth additional pertinent publications. Manual searches of relevant oncology journals and conference proceedings were also undertaken to ensure exhaustiveness of the literature review.

### 2.2 Inclusion and exclusion criteria

The inclusion criteria for this study were crafted with meticulous precision to guarantee a rigorous and targeted investigation. Specifically, studies were required to (1) studies that report the association between *CISD2* IHC expression and at least one of the predefined clinical pathological indicators (age, sex, depth of tumor invasion, lymphatic invasion status, TNM staging, histological grade) in GC patients; (2) study design with available full-text data, published in either Chinese or English; (3) immunohistochemistry was used to detect *CISD2* protein, with expression level assessed by a combined scoring system accounting for staining extent and intensity. The most significant finding was the identification of markedly dysregulated *CISD2* expression in GC patient tissues compared to non-GC controls. Conversely, explicit exclusion criteria were devised to remove investigations that could undermine the validity or pertinence of the analysis: studies addressing *CISD2* without relevance to GC; those with incomplete or inaccessible raw data pertaining to *CISD2* and GC; abstracts, case reports, and review articles; theses; animal-based research; meta-analyses; and redundant publications.

### 2.3 Literature screening and data extraction

Following the pre-set inclusion and exclusion criteria, two independent researchers undertook a meticulous literature screening process to gather pertinent materials, resolving any discrepancies or disagreements through collaborative discussions or by consulting a third, unbiased researcher. The extracted data encompassed a broad spectrum of information from the selected studies, including (1) foundational particulars such as the first author’s name, publication year, country of origin, and sample size; (2) the study designs that met the inclusion criteria; and (3) the outcome indicators along with their corresponding measurement data. In cases where the data was unpublished or insufficient, proactive steps were taken to contact the original authors to request additional or supplementary data, aiming to enrich the analysis and bolster the robustness of the findings, thereby demonstrating a steadfast commitment to upholding the utmost standards of data integrity and completeness in this study.

### 2.4 Quality evaluation of the included literature

Two independent reviewers used the Newcastle-Ottawa Scale (NOS) to assess the quality of the included studies (14–16). Whenever discrepancies or inconsistencies arose in their evaluations, the reviewers engaged in comprehensive discussions to arrive at a mutual agreement. According to NOS benchmarks, studies that achieve a score of at least six out of a possible nine points are deemed to be of outstanding quality. This rigorous evaluation protocol guaranteed that only studies adhering to stringent methodological criteria were included in the current analysis, thereby bolstering the credibility and dependability of the study’s conclusions.

### 2.5 Database validation and bioinformatics analysis

To evaluate *CISD2*’s impact on GC patients’ overall survival (OS), first progression survival (FP), and the human epidermal growth factor receptor 2 (HER2)-negative subtype survival outcome, we used the Kaplan-Meier plotter online database (https://kmplot.com/analysis/index.php?p=home) (17–21) for this independent exploratory bioinformatics analysis. Full methodological details of the analysis are explicitly reported below: (1) the specific *CISD2* probe ID used in this analysis is 244275_at; (2) the expression cutoff separating high and low *CISD2* mRNA expression groups was determined by the platform’s built-in auto-optimized cutoff algorithm; (3) the OS analysis included 631 GC patients, with a median follow-up duration of 40.7 months; the FP analysis included 522 GC patients; the *HER2*-negative subgroup survival analysis included 429 GC patients; (4) No formal statistical adjustment for multiple comparisons was performed across the three separate survival analyses, which represents a limitation of this exploratory analysis, as acknowledged in the subsequent limitations section.

### 2.6 Method of statistics

We used odds ratio (OR) as the effect size measure and performed all analyses in Stata 12.0, with 95% *CI* computed for each estimate. Heterogeneity was assessed via the Cochrane *Q* test (*α* = 0.1) (22). A fixed-effect model was applied when *P* > 0.1 and *I*² < 50% (23); otherwise, a random-effects model was adopted (24). When heterogeneity was detected, its sources were explored through sensitivity analysis.

## 3 Results

### 3.1 Literature screening process and results

The study initially sourced 708 publications across databases: 202 (PubMed), 200 (Web of Science), 26 (SinoMed), 128 (Wan Fang), 20 (VIP), 82 (Cochrane Library), and 50 (CNKI). Reviewing references yielded no additional literature. After rigorous screening, only three studies (8–10)—two English (8, 9) and one Chinese (10)—were included, as shown in **Figure 1**.

**Figure 1:**
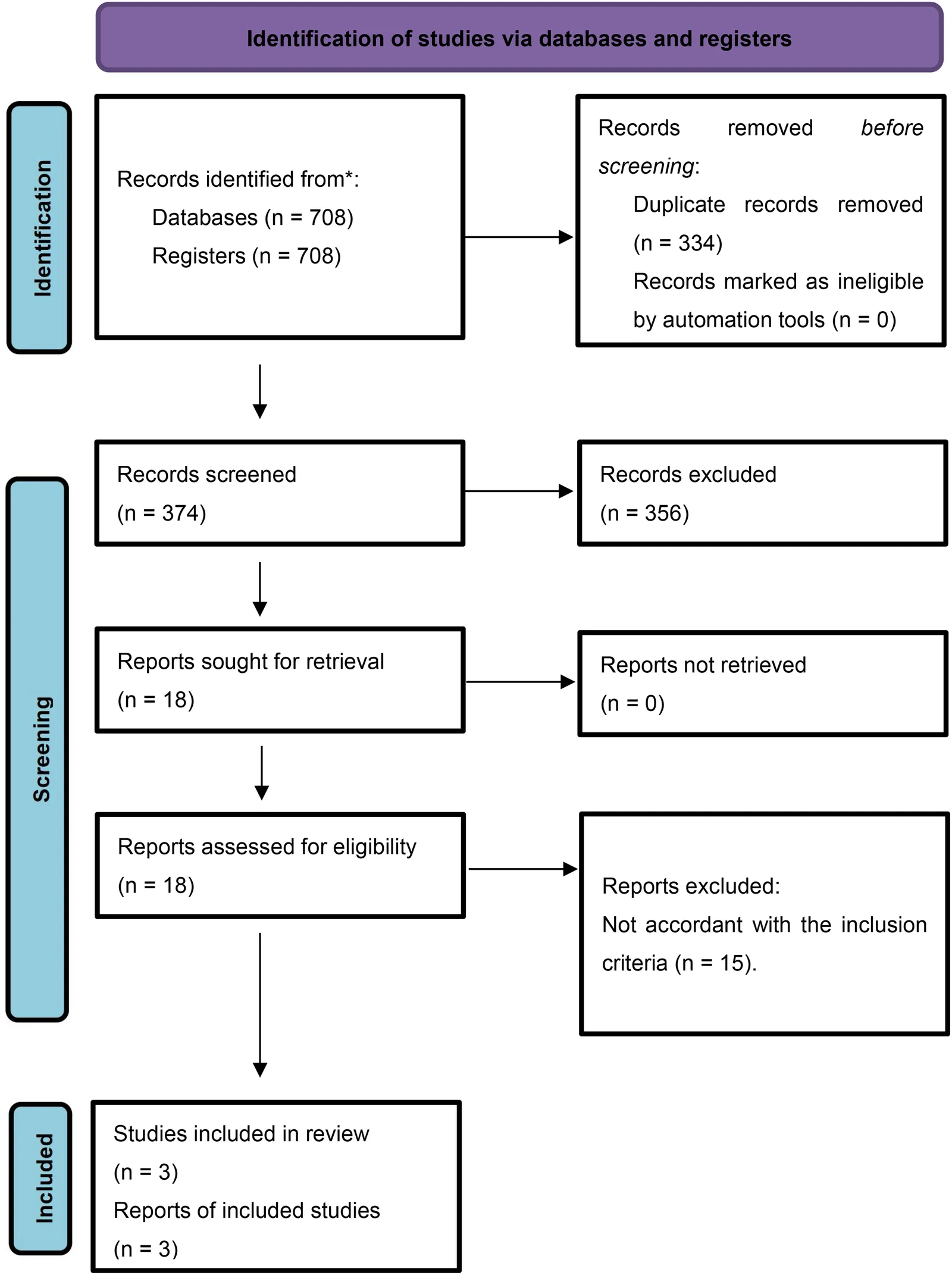
Literature screening process and outcomes.

### 3.2 Basic characteristics and quality evaluation results of the included literatures

The study’s final dataset included three publications (8–10) from 2015-2023, covering 1, 318 patients (**Table 1**). Using the NOS, all selected studies were confirmed to be of high quality (**Table 2**).

**Table 1:** Characteristics of included trials.

Author	Year	Country	Sample size (n=1318)		Research type	Method	IHC antibody
			Case group(n=764)	Control group(n=554)			
Wang Lan (8)	2015	China	210	–	Case-Control	IHC	Chicago (13318-1-AP)
Sun Yi (9)	2017	China	197	197	Case-Control	IHC	Chicago (13318-1-AP)
Zou Bingqing (10)	2023	China	357	357	Case-Control	IHC	Cell Signaling Technology (#60758)

IHC, Immunohistochemistry.

**Table 2:** Quality evaluation of included trials.

Author	Sample type	Tumor type	Expression	Factors	NOS score
Wang Lan (8)	Tissue	Gastric cancer	Increased	①②③④⑤⑥	8
Sun Yi (9)	Tissue	Gastric cancer	Decreased	①②③④⑤⑥	8
Zou Bingqing (10)	Tissue	Gastric cancer	Increased	①②③④⑤⑥	8

NOS, Newcastle-Ottawa Scale; ①age; ②sex; ③invasion depth; ④lymphatic invasion; ⑤TNM stage; ⑥histological grade.

### 3.3 Results of meta-analysis

#### 3.3.1 *CISD2* expression and age features

After assessing heterogeneity (*I*^2^ = 70.0%, *P* = 0.035) in three studies (8–10), we applied a random-effects model. The forest plot (**Figure 2A**) showed no significant group difference (*OR* = 1.37, 95%*CI*: 0.77-2.43, *P* = 0.286), indicating no notable decrease in *CISD2* expression related to GC patient age in the pooled analysis.

**Figure 2:**
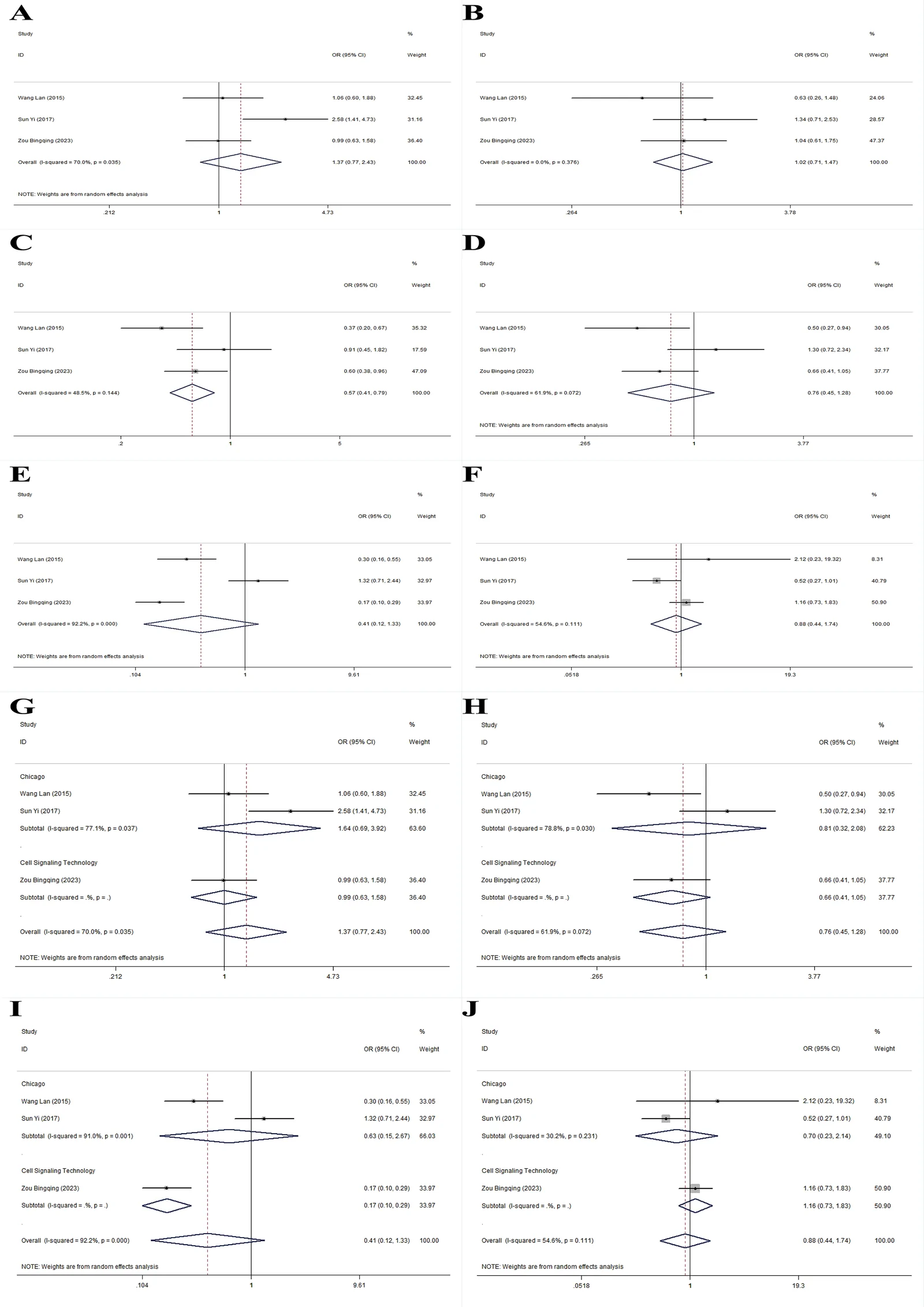
*CISD2* risk-clinicopathological links in gastric cancer: forest plots [**(A–F)**: Age, Sex, Invasion Depth, Lymphatic Invasion, TNM Stage, Histological Grade] and Subgroup Analyses [**(G–J)**: By Antibody Type].

#### 3.3.2 *CISD2* expression and sex features

After assessing low heterogeneity (*I*^2^ = 0.0%, *P* = 0.376) in three studies (8–10), we applied a fixed-effects model. The forest plot (**Figure 2B**) showed no significant group difference (*OR* = 1.02, 95%*CI*: 0.71-1.47, *P* = 0.897), indicating no notable sex-based risk reduction for *CISD2* in GC patients in the pooled analysis.

#### 3.3.3 *CISD2* expression and invasion depth features

Three studies (8–10) met the meta-analysis criteria, showing low heterogeneity (*I*^2^ = 48.5%, *P* = 0.144), prompting the use of a fixed-effects model. The results showed a notable group difference (*OR* = 0.57, 95%*CI*: 0.41-0.79, *P* = 0.001) (**Figure 2C**). The pooled analysis indicated that *CISD2* expression might decrease with increasing GC invasion depth.

#### 3.3.4 *CISD2* expression and lymphatic invasion features

The meta-analysis included three studies (8–10); due to heterogeneity (*I*^2^ = 61.9%, *P* = 0.072), a random-effects model was applied. The forest plot (**Figure 2D**) showed no significant group difference (*OR* = 0.76, 95%*CI*: 0.45-1.28, *P* = 0.295), indicating no clear link between lymphatic invasion and increased *CISD2* expression in GC patients in the pooled analysis.

#### 3.3.5 *CISD2* expression and TNM stage features

The meta-analysis, incorporating three articles (8–10), revealed high study heterogeneity (*I*^2^ = 92.0%, *P* = 0.000), necessitating a random-effects model. **Figure 2E** indicates no significant group difference (*OR* = 0.41, 95%*CI*: 0.12-1.33, *P* = 0.136), with pooled analysis showing no association between *CISD2* and TNM stage in GC patients.

#### 3.3.6 *CISD2* expression and histological grade features

The analysis included three articles (8–10). Due to study heterogeneity (*I*^2^ = 54.6%, *P* = 0.111), a random-effects model was used. Meta-analysis showed no significant group difference (*OR* = 0.88, 95%*CI*: 0.44-1.74, *P* = 0.711; **Figure 2F**), with no statistically significant risk reduction in *CISD2* and histological grade in GC patients.

### 3.4 Subgroup analyses

To explore heterogeneity sources and reduce their impact, we performed subgroup analyses by antibody type, age, lymphatic invasion, TNM stage, and histological grade. Results (**Figures 2G–J**) showed no significant heterogeneity between groups for these variables, indicating they did not substantially explain the observed study heterogeneity.

### 3.5 Sensitivity analyses

With merely three Chinese studies available (8–10), conventional publication bias tests were not applicable. Funnel plots are provided in **Figure 3** solely for illustrative purposes and were not subjected to statistical interpretation. Furthermore, sensitivity analysis (**Figure 4**) only showed that excluding any single study did not reverse the trend of pooled estimates, which does not constitute definitive evidence for the reliability of results.

**Figure 3:**
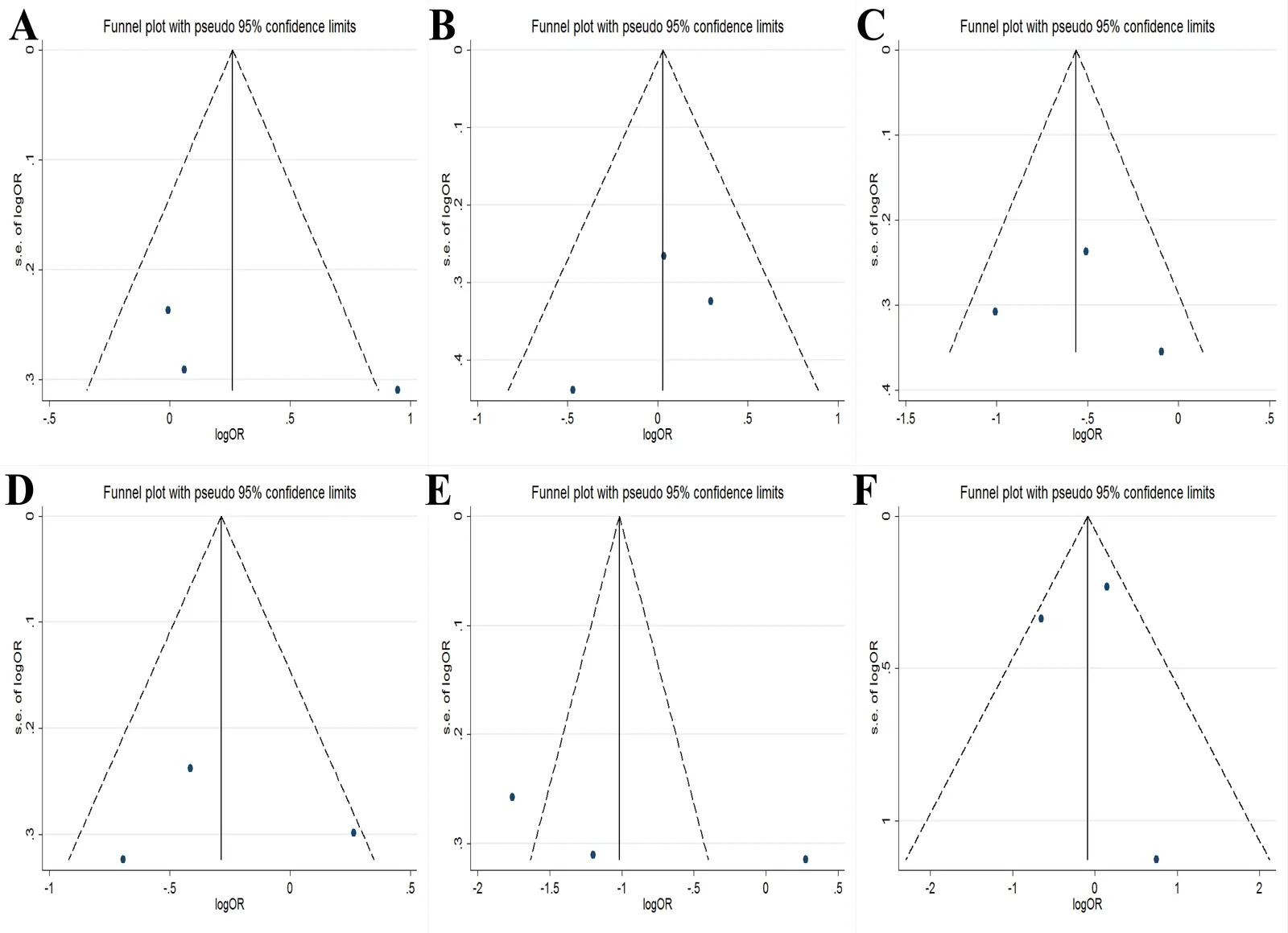
Funnel plots of *CISD2* risk vs. gastric cancer clinicopathological factors [**(A–F)**: Age, Sex, Invasion Depth, Lymphatic Invasion, TNM Stage, Histological Grade].

**Figure 4:**
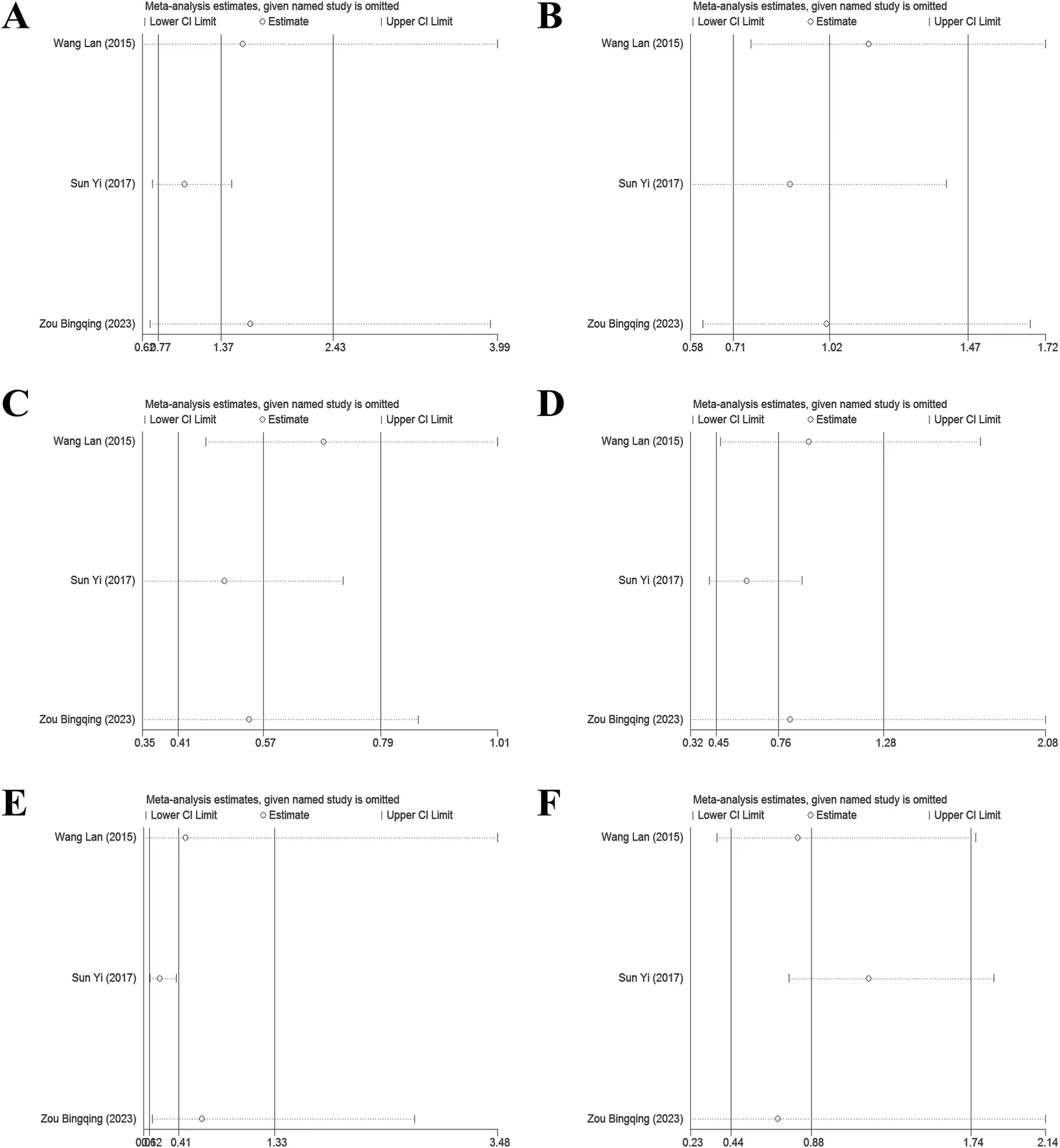
Sensitivity analyses of *CISD2* risk - clinicopathological parameter links in gastric cancer [**(A–F)**: Age, Sex, Invasion Depth, Lymphatic Invasion, TNM Stage, Histological Grade].

### 3.6 Database validation and bioinformatics analysis

Kaplan-Meier Plotter database interrogation demonstrated that elevated *CISD2* transcript levels were significantly associated with FP (*HR* = 1.3, *P* = 0.04), reduced OS (*HR* = 1.27, *P* = 0.041), and *HER2*-negative subtype (*HR* = 1.35, *P* = 0.034) in GC, suggesting that *CISD2* may serve as a potential prognostic biomarker in this malignancy (**Figure 5**).

**Figure 5:**
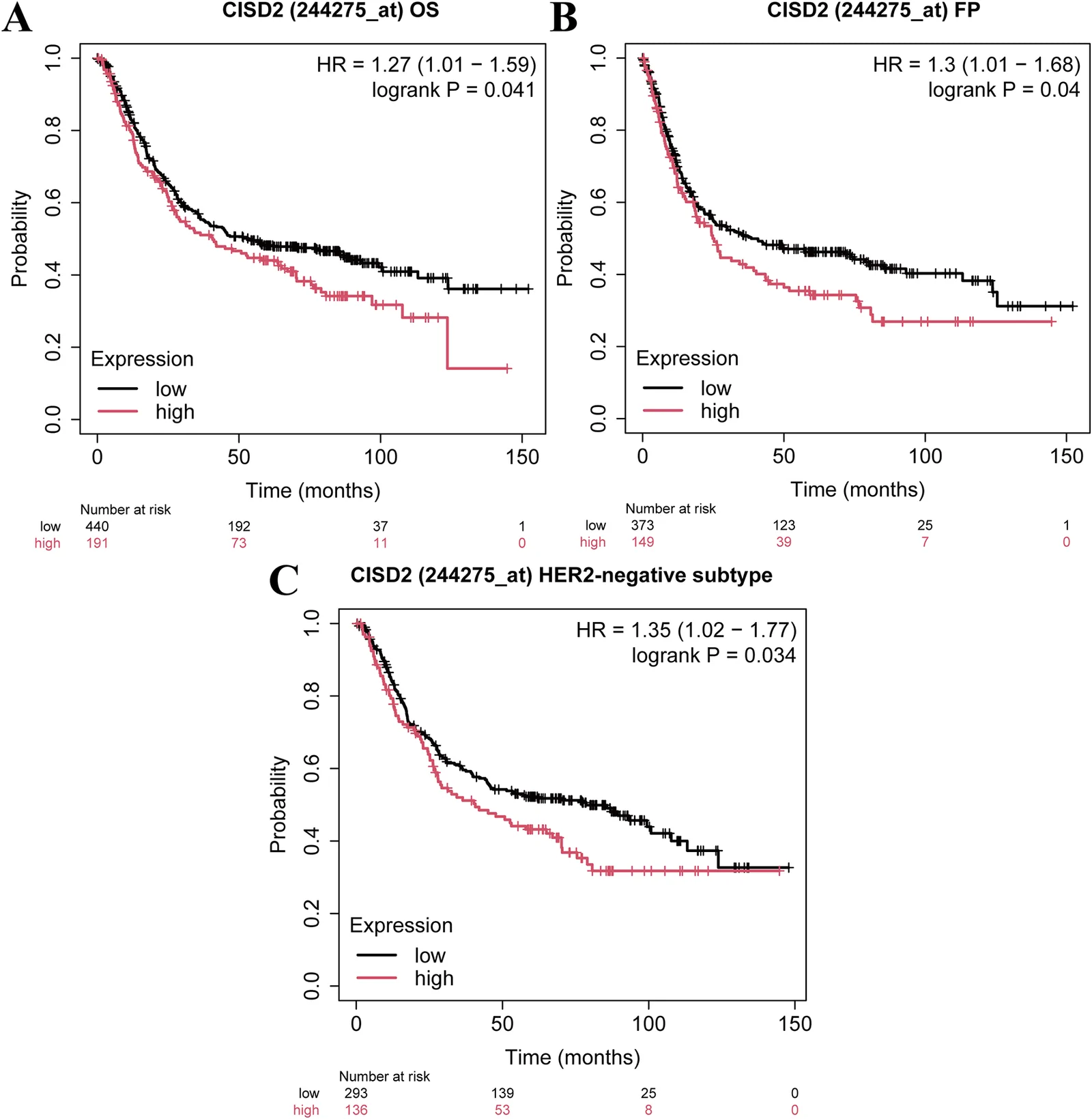
KM - plotter - Validated *CISD2* Expression’s Impact on Gastric Cancer Survival [**(A)**: OS; **(B)**: FP; **(C)**: HER2-negative subtype]: FP, First Progression Survival; OS, Overall Survival; HER2, Human Epidermal Growth Factor Receptor 2; KM-plotter, Kaplan Meier-plotter.

## 4 Discussion

The meta-analysis encompassed three studies, collectively comprising a total sample size of 1318 participants, all of which were conducted in Chinese populations. This geographic selection bias substantially limits the generalizability of our exploratory conclusions to non-Chinese, other ethnic and geographic populations, which should be taken into consideration when interpreting the results.

The exploratory meta-analysis results demonstrated no statistically significant correlation between *CISD2* protein expression in GC patients and age, sex, lymphatic invasion, TNM stage, or histological grade. A suggestive negative association was observed between *CISD2* IHC expression and tumor invasion depth (*OR* = 0.57), but this result should be interpreted with extreme caution, given that three included studies (8–10) show inconsistent trends. Specifically, Wang et al. (8) and Zou et al. (10) reported that elevated *CISD2* expression was associated with more advanced invasion and metastatic features, while Sun et al. (9) found no significant correlation between *CISD2* expression and infiltration depth. This apparent discrepancy originates from multiple potential sources: first, the IHC scoring thresholds for defining *CISD2* high/low expression are different across studies; second, two distinct antibodies were applied for IHC detection, which may have different binding affinity and detection sensitivity for *CISD2* protein; third, the distribution of T-stage subgroups in the three enrolled patient populations is heterogeneous. Given these inconsistencies, no definitive conclusions regarding the causal role of *CISD2* in regulating GC tumor invasion can be drawn from current limited evidence.

The independent Kaplan-Meier Plotter in silico analysis did not assess *CISD2* protein expression, but evaluated transcript-level *CISD2* mRNA abundance in GC tissues. This analysis cannot serve as direct confirmation of the protein-level meta-analysis findings. Instead, it provides separate, complementary prognostic evidence at the transcriptional dimension, which suggests that elevated *CISD2* mRNA expression correlates with poor survival outcomes in GC patients. Consistently, Wang et al. (8) also identified *CISD2* overexpression as an adverse prognostic marker in GC. Notably, *CISD2* functions context-dependently—it acts as a risk factor in untreated GC but may predict favorable response to 5-fluorouracil chemotherapy (9). Still, most existing studies, including those by Sun et al. (9) and Zou et al. (10), examined tissue *CISD2* primarily for diagnostic utility and overlooked its prognostic role in survival analysis. Taken together, *CISD2* appears to carry potential prognostic value in GC, but the underlying mechanisms still await further validation.

Several limitations of the present work should be fully acknowledged. The most critical concern stems from the limited number of eligible studies (n=3) incorporated into this meta-analysis, which carries important implications: (i) all pooled effect estimates in this exploratory study must be interpreted with extreme caution, given the inherent limited stability and precision of pooled results derived from only 3 datasets; (ii) the precision of corresponding confidence intervals may be compromised, especially for analyses with high heterogeneity (such as the TNM stage analysis with *I*²=92%); (iii) subgroup comparisons, funnel-plot evaluations of publication bias, and heterogeneity analyses may lack adequate statistical power, thereby raising the risk of spurious findings; and (iv) all three included studies were conducted in Chinese populations, which limits the external generalizability of conclusions to other ethnic groups. To address these shortcomings, subsequent large-scale meta-analyses incorporating more multinational independent studies are needed to corroborate these preliminary exploratory findings.

## 5 Conclusions

In summary, this exploratory hypothesis-generating meta-analysis, paired with independent transcript-level in silico complementary analysis, provides preliminary suggestive evidence that *CISD2* may be a candidate biomarker potentially associated with GC progression and prognosis. Further larger-scale prospective clinical studies and targeted mechanistic investigations are still indispensable to validate these associations before any clinical application of *CISD2* can be established.

## Data Availability

The original contributions presented in the study are included in the article/**Supplementary Material**. Further inquiries can be directed to the corresponding authors.
